# Predictive value of neutrophil to lymphocyte ratio for the clinical outcome of patients with ureteral stones: a systematic review and meta-analysis

**DOI:** 10.1186/s12894-025-02042-9

**Published:** 2026-01-21

**Authors:** Wei Chen, Zipei Cao

**Affiliations:** Department of Urology, Ningbo Urology and Nephrology Hospital, Ningbo, Zhejiang 315100 China

**Keywords:** Neutrophil, Lymphocyte, NLR, Ureteral stones, Meta-analysis

## Abstract

**Objectives:**

This study presents the first systematic review and meta-analysis assessing the predictive value of the neutrophil-to-lymphocyte ratio (NLR) on the clinical outcomes in individuals with ureteral stones.

**Methods:**

Relevant studies were systematically identified through searches in PubMed, Embase, Web of Science, and Cochrane, covering publications up to January 2025, focusing on studies that assessed the predictive role of NLR in the clinical outcomes of individuals with ureteral stones. Outcomes included spontaneous stone passage (SSP) and ureteric sepsis. Predictive data for SSP were derived from a cohort of patients receiving conservative medical treatment, while predictive data for ureteric sepsis were derived from a cohort of patients receiving surgical treatment. Sensitivity and subgroup analysis were conducted to evaluate the stability of the results and possible sources of heterogeneity.

**Results:**

10 cohort studies involving 4,859 patients with ureteral stones were analyzed in this meta-analysis. The meta-analysis of multivariate data showed that SSP rate was significantly lower in the high NLR group compared to the low NLR group (OR: 0.46; 95% CI: 0.33, 0.64; *P* <0.00001), and the risk of ureteric sepsis in the high NLR group was significantly higher than that in the low NLR group (OR: 2.50; 95% CI: 1.31, 4.76; *P* = 0.005). Subgroup analysis suggested that the prognostic value of NLR for patients with ureteral calculi was not affected by stone size and NLR cut-off value.

**Conclusions:**

NLR can be used as an independent predictor of the SSP and the risk of ureteric sepsis in patients with ureteral stones. Future large-scale, multicenter, prospective clinical studies are necessary to further confirm the relationship between NLR and the clinical outcomes of patients with ureteral stones.

**Clinical trial number:**

Not applicable.

**Supplementary Information:**

The online version contains supplementary material available at 10.1186/s12894-025-02042-9.

## Introduction

Urinary tract stones are common condition in urology, including stones occurring in any anatomical location of the urinary system. The incidence in Europe and the United States is as high as 8.8%, and in China it is 1% to 5%. In southern China, influenced by diet and climate, the incidence rate of urinary tract stones is as high as 5% to 10% [1, 2]. Due to the narrow lumen of the ureter, ureteral stones are likely to cause obstruction. After obstruction occurs, the speed of urine flushing the ureter slows down, creating suitable conditions for the aggregation and reproduction of various bacteria above the obstruction site, which in turn makes ureteral stones increasing the risk of urinary tract infections [3].

The high economic burden and increasing morbidity have prompted a cautious management strategy for ureteral stones, including the timing of intervention and implementation of medical expulsion therapy [4]. As most stones smaller than 10 mm pass on their own, interventional treatment of ureteral stones may be considered overtreatment and result in unnecessary costs [5]. However, wait-and-see treatment may lead to unexpected complications, such as renal failure and urosepsis [6]. As a result, selecting the right patients is essential for the appropriate management of ureteral stones. Multiple studies have sought to identify patients with ureteral stones who are suitable candidates for watchful waiting [7]. Evidence suggests that spontaneous stone expulsion is more likely in cases involving smaller stones, distal ureteral location, and lower levels of inflammatory markers, such as white blood cell count, neutrophil count, and C-reactive protein [8, 9]. Elevated inflammatory markers may reflect ureteral stone obstruction.

Conversely, ureteral sepsis is a systemic septic reaction caused by infection following obstruction from urinary tract stones. It is a serious complication that can progress rapidly and be life-threatening [10]. It is difficult to achieve ideal treatment effects with antibiotics alone, and the risk of lithotripsy for such patients is relatively high [11]. If ureteral calculi with concomitant ureteric sepsis are not promptly identified and treated, persistent obstruction can increase the risk of septic shock, significantly raising patient mortality [12]. Therefore, early detection and diagnosis of patients with ureteral calculi combined with urinary sepsis is of great significance. Numerous studies have indicated a potential relation between inflammatory status and the risk of ureteric sepsis in individuals with ureteral calculi [13, 14].

Among the various inflammatory markers studied, the neutrophil-to-lymphocyte ratio (NLR) is a simple marker that reveals differences in inflammatory cells. It has been extensively investigated in various cancers and infectious diseases. The higher the ratio, the worse the overall disease and prognosis [15–18]. Moreover, numerous analyses have highlighted the predictive value of NLR for the clinical outcomes of patients with ureteral stones (mainly including spontaneous stone passage (SSP) and ureteric sepsis) [13, 14, 19–26]. However, due to differences in research cycles, stone size, treatment methods, NLR cut-off values, etc., there is no conclusive evidence whether NLR can effectively predict the clinical outcomes of ureteral stones. Therefore, we conducted this analysis is to assess the clinical value of NLR in predicting the clinical outcomes of ureteral stones for the first time through systematic literature retrieval and meta-analysis. Thus offering the most extensive evidence-based basis for constructing a precise prognostic model for ureteral stone outcomes.

## Methods

### Literature search

Following PRISMA 2020 guidelines (Preferred Reporting Items for Systematic Reviews and Meta-Analyses) [27], this analysis was prospectively registered in PROSPERO (CRD420251002028). We systematically searched PubMed, Embase, Web of Science, and Cochrane for studies published up to January 2025 that assessed the predictive value of the NLR in relation to the clinical outcomes of patients with ureteral stones. The literature search was conducted using the following terms: “Neutrophils”, “Lymphocytes”, and “Ureteral Calculi”. The specific search strategy in PubMed is as follows: ((((“Neutrophils“[Mesh]) OR (((Neutrophil) OR (LE Cells)) OR (LE Cell))) AND ((“Lymphocytes“[Mesh]) OR (((Lymphocyte) OR (Lymphoid Cells)) OR (Lymphoid Cell)))) AND (ratio)) AND ((“Ureteral Calculi“[Mesh]) OR (((Ureteral Calculus) OR (Ureterolithiasis)) OR (Ureteral stone))). Next, we manually screened of the reference lists from all selected analysis. Two researchers independently conducted the retrieval and evaluation of relevant articles. Any differences arising during the selection process were addressed through discussion. A comprehensive overview of the literature search strategy is provided in Table S1.

### Inclusion and exclusion criteria

Eligibility criteria for studies included the following conditions: (1) the study was a cohort study, RCT, or case-control study; (2) the analysis was conducted on patients with ureteral stones; (3) the study assessed the predictive value of the NLR in relation to clinical outcomes in patients with ureteral stones; (4) at least one survival outcome (SSP, ureteric sepsis, etc.) was analyzed; (5) complete data to analyse multivariate data of odds ratio (OR) with 95% confidence interval (CI). Excluded from the analysis were study protocols, unpublished studies, non-original studies (letters, comments, abstracts, correction, and replies), studies without sufficient data, and review articles.

### Data abstraction

Data abstraction was independently performed by two authors. Any discrepancies were addressed by discussion. The extracted information from eligible studies included the following: first author’s name, year of publication, country of study, study design, research population, sample size, age, gender, stone size, NLR cut-off, and odds ratio (OR) from multivariate analysis for SSP and ureteric sepsis. Predictive data for SSP were derived from a cohort of patients receiving conservative medical treatment, while predictive data for ureteric sepsis were derived from a cohort of patients receiving surgical treatment. In cases of insufficient research data, corresponding authors were reached out to request complete datasets, if accessible.

### Quality evaluation

The quality of the included cohort studies was conducted using the Newcastle-Ottawa Scale (NOS) [27], categorizing studies with scores between 7 and 9 as high quality [28]. Studies scoring below 7 were excluded from quantitative analysis. Two authors independently evaluated the quality of all included studies, resolving any disagreements through discussion.

### Statistical analysis

Analysis was conducted using Review Manager 5.4.1. OR was employed for data synthesis, with findings reported as 95% confidence intervals (CIs). The chi-squared (χ²) test (Cochran’s Q) and the I² index were used to assess heterogeneity across outcomes [29]. High heterogeneity was defined as a χ² P-value of less than 0.1 or an I² value greater than 50%. A random-effects model was applied to estimate the overall OR for each outcome. Additionally, a sensitivity analysis was conducted to evaluate the influence of each incorporated analysis on the total OR for outcomes with at least three studies. Subgroup analyses were conducted for eligible outcomes based on stone size and NLR cut-off values to assess the stability of results and identify factors influencing heterogeneity. Furthermore, possible risk of publication bias was evaluated using funnel plots generated in Review Manager 5.4.1 and Egger’s regression tests [30] performed in Stata 15.1 (Stata Corp, College Station, Texas, USA) for outcomes with at least three included studies. A P-value below 0.1 was regarded as evidence of significant publication bias.

## Results

### Literature retrieval, study characteristics, and baseline

**Fig. 1:**
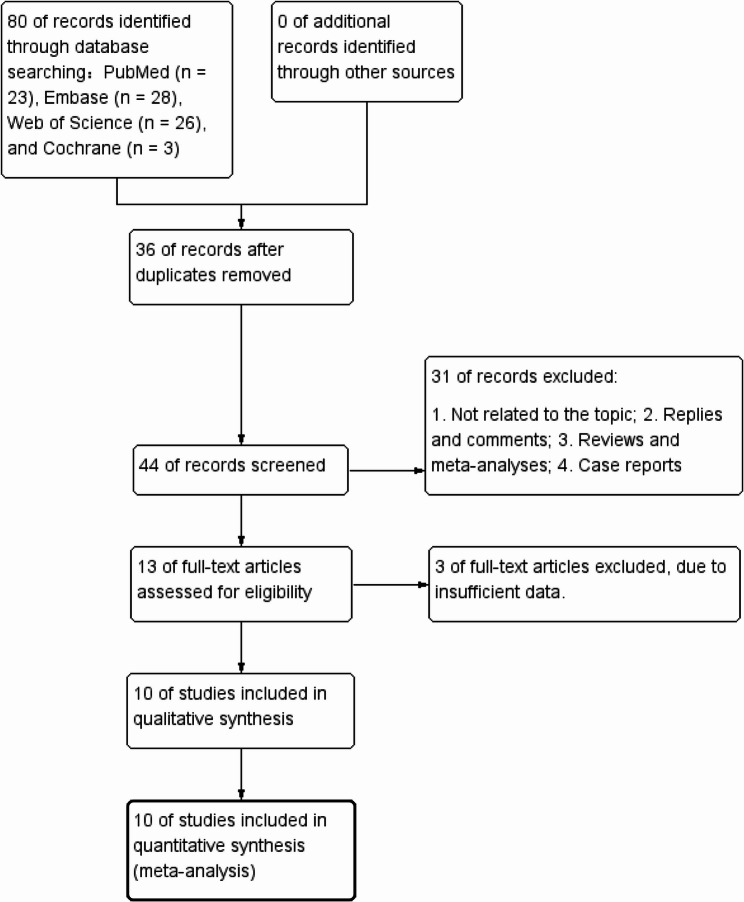
Flowchart of the systematic search and selection process

Figure 1 presents the flowchart outlining the literature retrieval and selection process. 80 relevant studies were identified across PubMed (*n* = 23), Embase (*n* = 28), Web of Science (*n* = 26), and Cochrane (*n* = 3). After removal of duplicate studies, 44 titles and abstracts were reviewed for eligibility. Finally, 10 cohort studies comprising 4,859 patients were included in this analysis [13, 14, 19–26]. 12 comparison groups were extracted from 10 studies. Of these, eight studies examined SSP in patients with ureteral stones treated with medical therapy [19–26], and two studies [13, 14] reported on the incidence of ureteric sepsis after surgical treatment. Table 1 summarizes the characteristics and quality assessment of included cohort studies.

**Table 1:** Characterisitic and quality evaluation of included studies

Study	Country	Study design	Population	No. of patients	Gender		Mean/median age	Mean/median stone size (mm)	NLR cut-off	NOS score
					Male	Female				
Abou Heidar 2020a	USA	Cohort	Ureteral stone of less than 10 mm	619	442	177	46.8	5	1.9	8
Abou Heidar 2020b	USA	Cohort	Ureteral stone of less than 10 mm	619	442	177	46.8	5	2.87	8
Abou Heidar 2020c	USA	Cohort	Ureteral stone of less than 10 mm	619	442	177	46.8	5	4.87	8
Abou Heidar 2022-I	USA	Cohort	Ureteral stone less than 10 mm	1186	868	318	45	5	3.14	9
Abou Heidar 2022-II	USA	Cohort	Patients discharged from emergency department on conservative treatment for ureteral stone (less than 10 mm)	450	340	110	44	5.2	3.14	7
Can Cilesiz 2020	Turkey	Cohort	Patients with a single distal ureteral stone between 5 and 10 mm in diameter and no indications for interventional therapy	54	38	16	37.1	NA	NA	9
Coskun 2022	Turkey	Cohort	Patients aged 18–55 who were referred with middle and distal ureter ureteral stones between 5–7 mm	100	50	50	41.5	6.55	NA	8
Hasan 2024	India	Cohort	Patients who underwent medical expulsive therapy for ureteric stones	156	90	66	NA	7.8	NA	7
Lee 2017	Korea	Cohort	Patients diagnosed with unilateral ureteral stones	113	77	36	52.6	4.3	2.3	7
Li 2024	China	Cohort	Patients with ureteral stones who underwent surgical treatment	520	234	286	NA	10.2	3.53	8
Selvi 2021	Turkey	Cohort	Patients with solitary unilateral ureteral stones less than 10 mm in diameter	280	152	128	44.5	5	1.96	7
Villanueva-Congote 2024	USA	Cohort	Patients diagnosed with obstructing ureteral stones who underwent urgent stent placement	143	56	87	61	NA	10	8

### SSP

**Fig. 2:**
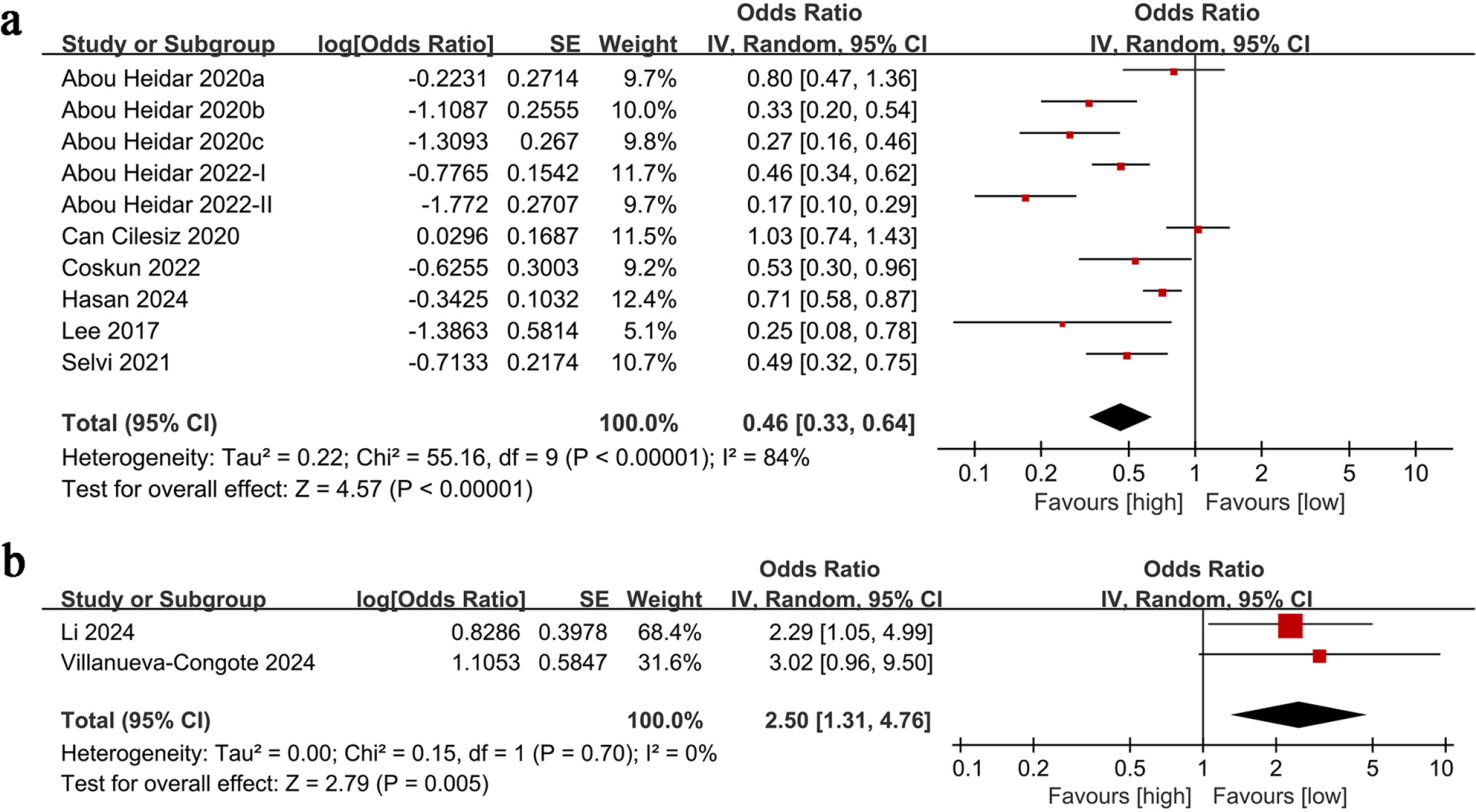
Forest plots of the association between NLR and SSP (**A**), ureteric sepsis (**B**)

The SSP results were pooled from 8 cohort studies (10 comparison groups) [19–26], and meta-analysis of multivariate data revealed a notably lower SSP rate in the group with increased NLR in comparison with the group with low NLR (OR: 0.46; 95% CI: 0.33, 0.64; *P* <0.00001). Significant heterogeneity was detected (*I*^2^ = 84%, *P* <0.00001) (Fig. 2A).

**Fig. 3:**
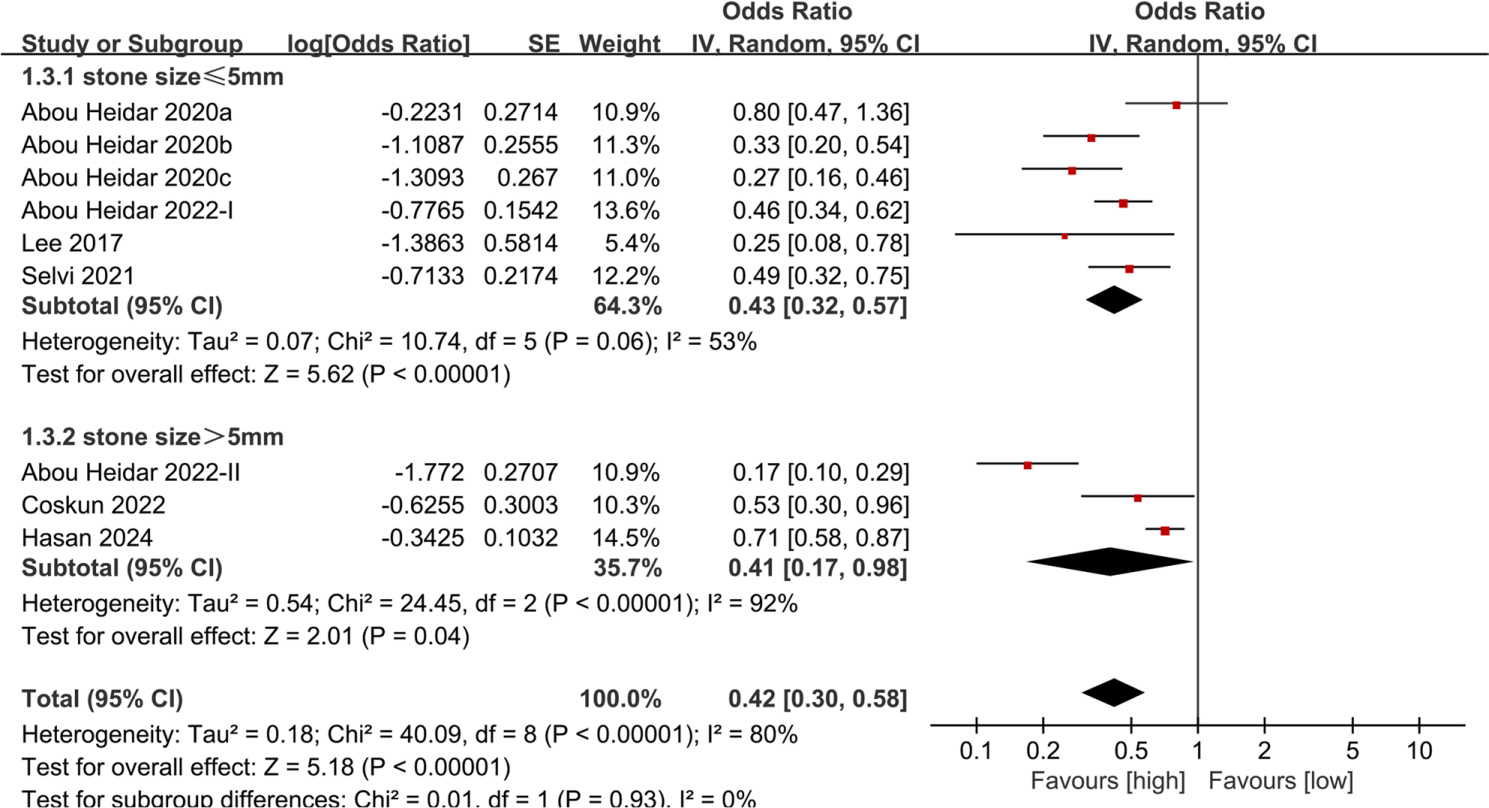
Subgroup analysis of the association between NLR and SSP based on stone size

To assess the stability of the results and identify possible contributors to heterogeneity, a subgroup analysis was initially performed based on stone size. The results showed that in the subgroups with stone size ≤ 5 mm (OR: 0.43; 95% CI: 0.32, 0.57; *P* <0.00001) and > 5 mm (OR: 0.42; 95% CI: 0.30, 0.58; *P* = 0.04), the predictive value of NLR for SSP was still significant, but the overall heterogeneity decreased significantly in ≤ 5 mm subgroup (53%) (Fig. 3).

**Fig. 4:**
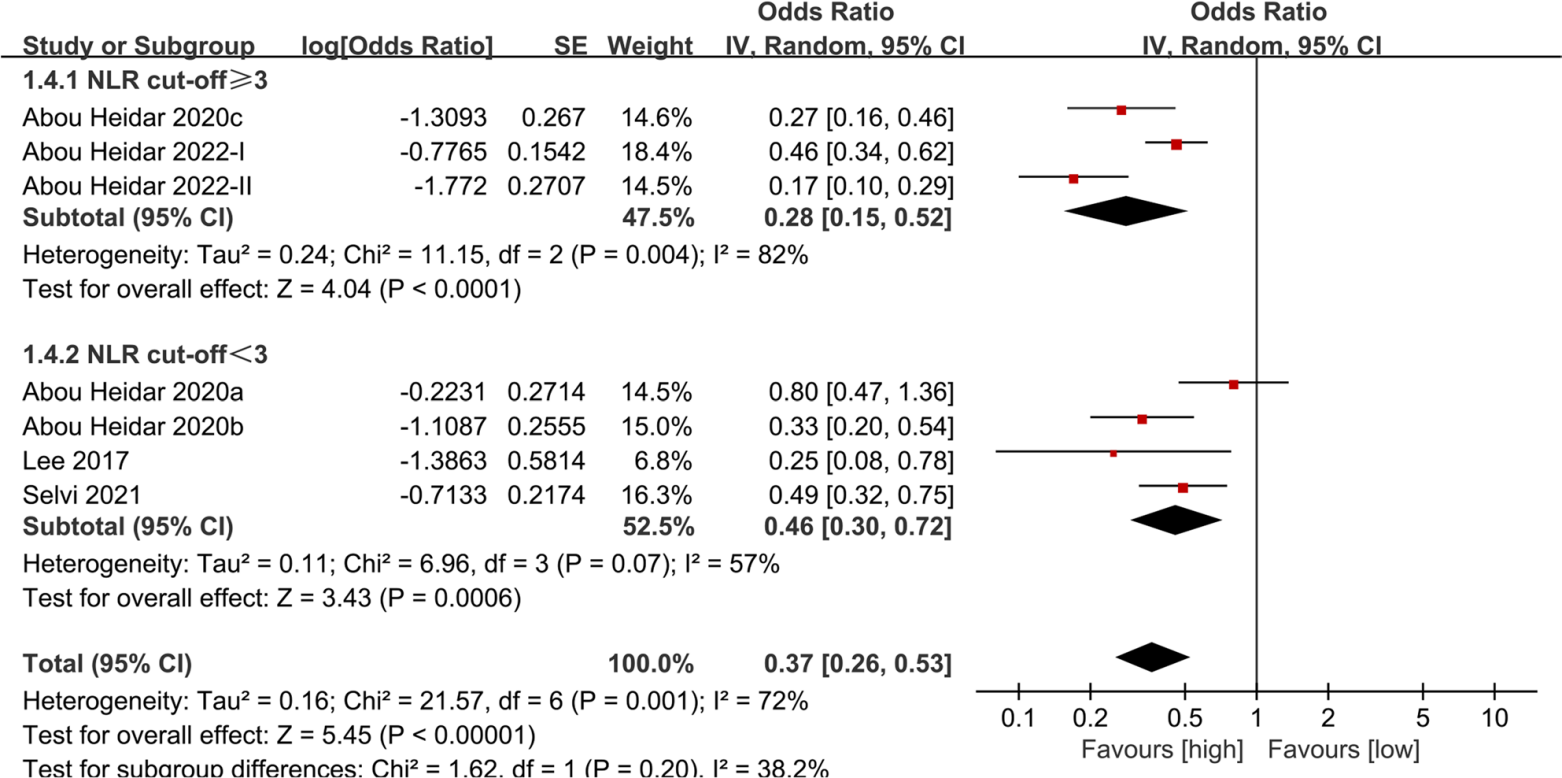
Subgroup analysis of the association between NLR and SSP based on NLR cut-off

Next, we conducted subgroup analysis according to the NLR cut-off value. The results showed that the predictive value of NLR for SSP was still significant in the subgroups with cut-off values ​​≥3 (OR: 0.28; 95% CI: 0.15, 0.52; *P* <0.0001) and < 3 (OR: 0.46; 95% CI: 0.30, 0.72; *P* = 0.0006), but the overall heterogeneity was significantly reduced in the subgroup with cut-off values ​​<3 (57%) (Fig. 4).

### Ureteric sepsis

Results of ureteric sepsis were pooled from 2 cohort studies [13, 14], and meta-analysis of multivariate data revealed a notably higher risk of ureteric sepsis in the group with high NLR compared with the group with low NLR (OR: 2.50; 95% CI: 1.31, 4.76; *P* = 0.005). No significant heterogeneity was observed (*I*^2^ = 0%, *P* = 0.70) (Fig. 2B).

### Publication bias and sensitivity analysis

**Fig. 5:**
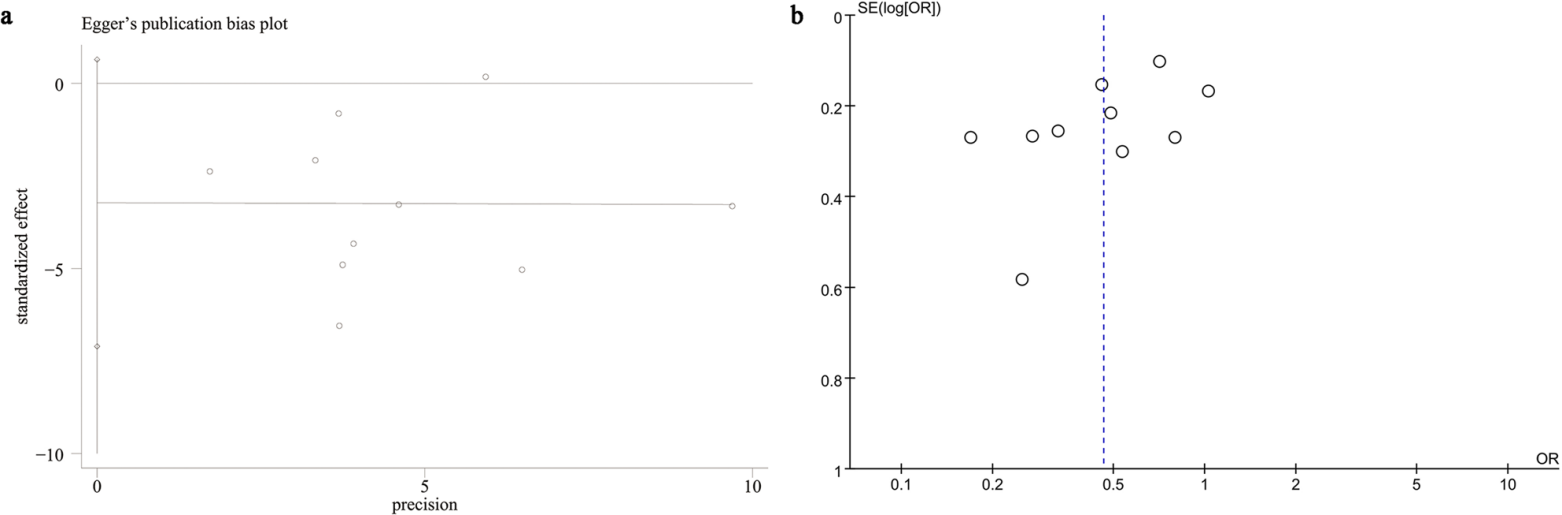
Egger’s test plot (**A**) and funnel plot (**B**) of SSP

**Fig. 6:**
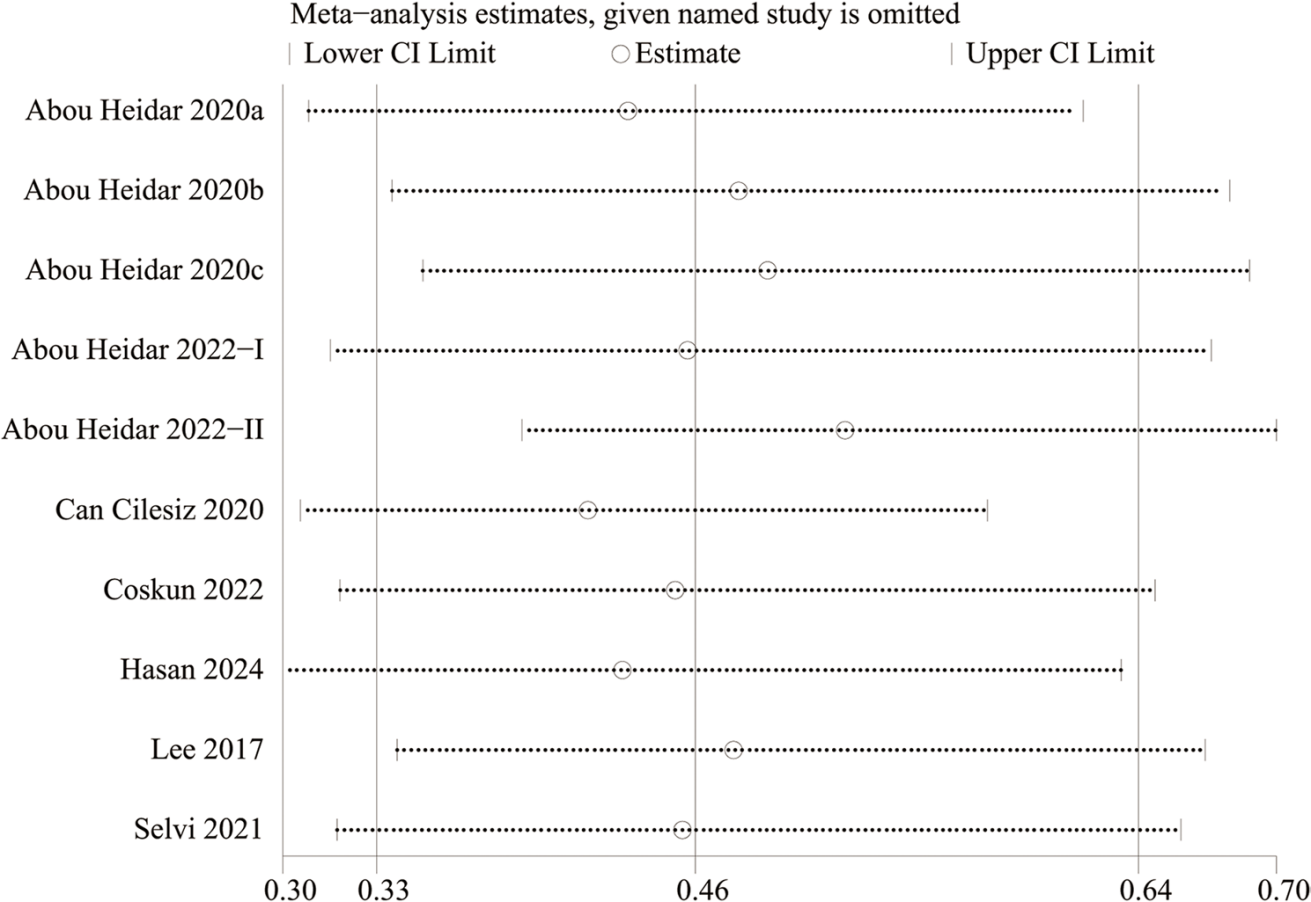
Sensitivity analysis of the association between NLR and SSP

We analyzed the possible risk of publication bias through funnel plots and Egger’s regression tests for SSP. Egger’s test (*P* = 0.091) (Fig. 5A) and funnel plots (Fig. 5B) did not detect a significant publication bias for SSP. Additionally, a sensitivity analysis was conducted on the SSP results to evaluate the impact of each cohort study on the overall OR by sequentially excluding individual articles. The analysis revealed that the overall OR remained stable after the removal of each cohort study for SSP (Fig. 6).

## Discussion

There are multiple treatment strategies for ureteral stones, mainly including expectant management (medical therapy), endoscopic surgery, or shock wave lithotripsy (SWL) [31]. Medical therapy is a cost-effective and non-invasive approach; however, it is associated with stone-specific risks, including urinary tract infections, kidney damage, and patient discomfort or pain. In contrast, stone removal through SWL is safer than medical expulsion therapy and has a higher stone-free rate. However, these procedures are expensive and may be associated with procedure-specific complications, including urinary tract infection, hematoma formation, and urinary extravasation [32]. In addition, postponing surgical treatment until conservative treatment fails places a significant burden on patients and increases the cost of treating stones compared with immediate surgical management [33]. Thus, informed clinical decision-making, grounded in evidence, is crucial yet challenging in the treatment of ureteral stones. This uncertainty has driven researchers to identify more accurate and effective markers that can assist urologists in accurately categorizing patients, to enable them to adopt the best management strategy to improve patient outcomes and reduce treatment risks and costs.

In this analysis, we assessed the predictive value of NLR for SSP and ureteric sepsis in patients with ureteral calculi. Results demonstrated a significantly reduced SSP rate in the high NLR group compared to the low NLR group. Additionally, a higher NLR was associated with a substantially greater risk of ureteric sepsis. These findings suggest that NLR has predictive value for clinical outcomes in individuals with ureteral calculi and should be considered in clinical decision-making and treatment strategies. Our findings are consistent with the majority of previously published studies [19, 20, 24]. In addition, the sensitivity analysis of this study did not find a significant impact of a single study on the overall findings, and no significant publication bias was observed, suggesting that the evidence from this study is reliable. Furthermore, this study performed subgroup analysis based on stone size and NLR cut-off value, and the results suggested that the prognostic value of NLR for individuals with ureteral calculi was not affected by stone size and NLR cut-off value. However, it is worth noting that the heterogeneous subgroup analysis suggested that both stone size and NLR cut-off value were sources of significant heterogeneity in SSP. This result suggests that future studies need to include more patients with ureteral stones of different diameters and adopt diversified NLR grouping schemes to further clarify the optimal conditions for the use of NLR.

As an inflammatory marker, NLR has shown certain predictive value in many diseases. In ureteral stones, the increase in NLR may be related to inflammation of the ureteral wall caused by stones. This inflammation may cause spasm and edema of the ureteral smooth muscle, thereby affecting the discharge of stones [34, 35]. Therefore, NLR may become a potential indicator for predicting spontaneous stone discharge in ureteral stones. In addition, NLR can also be detected in combination with other inflammatory markers including C-reactive protein (CRP) and serum procalcitonin (PCT) to improve the accuracy of prediction. Studies have shown that the area under the receiver operating curve (AUC) of the combined detection of CRP, PCT and NLR is significantly higher than that of a single indicator, showing higher sensitivity and specificity [13]. This means that by jointly detecting these inflammatory indicators, we can more accurately predict the spontaneous stone discharge in patients with ureteral stones.

However, it should be noted that although NLR shows certain application value in predicting the spontaneous excretion of ureteral stones, its predictive ability may be affected by many factors. For example, the patient’s age, gender, underlying diseases, etc. may affect the NLR level, thereby affecting the accuracy of its prediction [36]. Therefore, in practical applications, we need to comprehensively consider the patient’s clinical condition and combine other imaging and laboratory test results to make more comprehensive treatment decisions. In addition to inflammatory indicators such as NLR, there are some other factors that may also affect the spontaneous excretion of ureteral stones. For example, the physical characteristics of the stone, including composition, morphology, and surface roughness, along with patient’s eating habits, exercise volume and other living habits may affect the stone excretion process [13, 14]. Therefore, when predicting the spontaneous excretion of ureteral stones, we need to comprehensively consider multiple factors to improve the accuracy and reliability of the prediction.

On the other hand, studies have shown that the normal range of NLR for adults is between 0.8 and 2.2. When the value is higher than 3.0 or lower than 0.7, it indicates pathological changes in the body, such as malignant tumors, coronary heart disease, inflammation, and mental illness. When the value is between 2.3 and 3.0, it is necessary to be highly vigilant about the occurrence of the above diseases. For patients with sepsis, when the NLR value is greater than 7.0, the mortality rate of patients is significantly increased [37]. Recently, some scholars have found that elevated NLR has early warning value for ureteral stones combined with urinary sepsis, and its value changes synchronously with the severity of infection. Compared with the percentage of neutrophils, NLR has higher sensitivity and specificity, which also supports the conclusion of this study [13, 14]. Therefore, based on inflammatory indicators such as NLR, it is effective to warn patients with ureteral stones about the occurrence of ureteral sepsis, which is helpful for early active clinical intervention and has important significance for improving the survival rate of patients.

The findings of this study emphasize the clinical relevance of NLR, a widely adopted hematological index, in predicting the prognosis of individuals with ureteral stones.

However, certain limitations remain. First, there is no unified standard for the selection and calculation of the optimal cut-off value of NLR. The ROC curve method and the median method are frequently applied in statistics. Due to differences in the methods used to determine the NLR cut-off value in the analyzed literature, the number of cases and the NLR cut-off value are quite different, resulting in certain heterogeneity in the results. Secondly, while this study included individuals with ureteral stones, there were differences in the basic characteristics, stone size, and treatment methods of the patients included in each study, which may lead to unavoidable heterogeneity. Furthermore, the data reporting on the specific time to SSP or the time of intervention in the original studies included were inconsistent and incomplete. Therefore, this meta-analysis could not quantitatively combine these time indicators as outcomes, which is another limitation of this study. Future research should focus on and standardize the reporting time-related indicators to provide richer prognostic information. In addition, the studies incorporated in this meta-analysis had a limited sample size, with most of the data originating from single-center studies conducted in American and Asian countries, which inevitably led to a certain degree of selection bias in this study. Despite these limitations, this analysis is the first evidence-based study reporting the predictive value of NLR for the risk of SSP and ureteric sepsis in patients with ureteral stones. The results of this study support the idea that in the clinical treatment of individuals with ureteral stones, we should focus on the changes in NLR levels and establish a more effective predictive model incorporating NLR and other relevant factors to enhance ureteral stone prognosis and reduce treatment risks and costs.

## Conclusion

The multivariate data meta-analysis showed that NLR can serve as an independent predictor of SSP occurrence and ureteric sepsis risk in individuals with ureteral stones. A higher NLR correlated with a lower SSP rate and an increased risk of ureteric sepsis. Given the limitations of a small sample size and unavoidable heterogeneity, further validation of the relationship between NLR and clinical outcomes in ureteral stone patients necessitates large-scale, multicenter, prospective clinical studies.

## Supplementary Information

Supplementary Material 1.

## Data Availability

The data used to support the findings of this study are included within the article.
