# Supplementary material for: Predictive value of neutrophil to lymphocyte ratio for the clinical outcome of patients with ureteral stones: a systematic review and meta-analysis
Source: BMC Urol. 2026 Jan 21;26:47. doi: 10.1186/s12894-025-02042-9 (PMC12908324; doi:10.1186/s12894-025-02042-9)
Supplement: Supplementary file 1 — Supplementary Material 1. [file 12894_2025_2042_MOESM1_ESM.docx]

TableS1 Literature Search Strategy

Pubmed-23

(((("Neutrophils"[Mesh]) OR (((Neutrophil) OR (LE Cells)) OR (LE Cell))) AND (("Lymphocytes"[Mesh]) OR (((Lymphocyte) OR (Lymphoid Cells)) OR (Lymphoid Cell)))) AND (ratio)) AND (("Ureteral Calculi"[Mesh]) OR (((Ureteral Calculus) OR (Ureterolithiasis)) OR (Ureteral stone)))

Embase-28


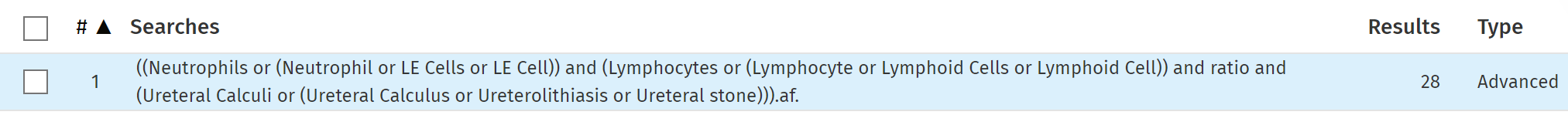


Cochrane-3


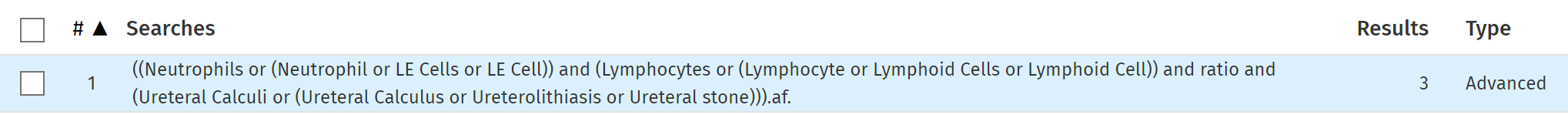


WOS-26

((((Neutrophils) OR (((Neutrophil) OR (LE Cells)) OR (LE Cell))) AND ((Lymphocytes) OR (((Lymphocyte) OR (Lymphoid Cells)) OR (Lymphoid Cell)))) AND (ratio)) AND ((Ureteral Calculi) OR (((Ureteral Calculus) OR (Ureterolithiasis)) OR (Ureteral stone))) (All Fields)
